# Traction Machine State Recognition Method Based on DPCA Algorithm and Convolution Neural Network

**DOI:** 10.3390/s23146646

**Published:** 2023-07-24

**Authors:** Dongyang Li, Jianyi Yang, Zaisheng Pan, Nanyang Li

**Affiliations:** 1 College of Information Science and Electronic Engineering, Zhejiang University, Hangzhou 310013, China; 2 Hangzhou Special Equipment Inspection and Research Institute, Hangzhou 310051, China; 3 Institute of Cyber-Systems and Control, Zhejiang University, Hangzhou 310027, China; 4 Zhejiang Promotion Association for Intelligent Technology Standards Innovation, Hangzhou 311121, China

**Keywords:** machinery industry, traction machine, convolution neural network, Fourier transform, status identification

## Abstract

It is important to improve the identification accuracy of the operating status of elevator traction machines. The distribution difference of the time-frequency signals utilized to identify operating circumstances is modest, making it difficult to extract features from the vibration signals of traction machines under various operating conditions, leading to low recognition accuracy. A novel method for identifying the operating status of traction machines based on signal demodulation method and convolutional neural network (CNN) is proposed. The original vibration time-frequency signals are demodulated by the demodulation method based on time-frequency analysis and principal component analysis (DPCA). Firstly, the signal demodulation method based on principal component analysis is used to extract the modulation features of the experimentally measured vibration signals. Then, The CNN is used for feature vector extraction, and the training model is obtained through multiple iterations to achieve automatic recognition of the running state. The experimental results show that the proposed method can effectively extract feature parameters under different states. The diagnostic accuracy is up to 96.94%, which is about 16.61% higher than conventional methods. It provides a feasible solution for identifying the operating status of elevator traction machines.

## 1. Introduction

In recent years, with the widespread adoption of elevators in mid to high-rise buildings, traction machines, which serve as vital power output and transmission equipment in elevator systems, have garnered increasing attention [1]. Elevator malfunctions have emerged as a significant latent risk in peoples’ lives, making the enhancement of elevator safety performance a pivotal concern for the elevator industry [2]. Abundant experimental data substantiates that promptly comprehending changes in the operational status of traction machines and detecting operational abnormalities at an early stage hold tremendous significance in preventing equipment failures [3]. Currently, the research on non-destructive testing methods for elevator traction machines remains limited, primarily focusing on the analysis of noise and vibration frequency to ascertain the normality of their operational status. When abnormal and severe noise and vibration frequencies occur, the traction machine often experiences critical faults. In contrast to noise monitoring, vibration monitoring offers the advantages of long-term surveillance, ease in determining operating conditions, and lower costs.

Numerous scholars have conducted extensive research on the discrimination of the operating status of traction machines. Jiahui Tang et al. [4] proposed a method based on a mixed pool deep trust network (MP-DBN), which has a good feature extraction ability and diagnostic efficiency, but the model does not have an excellent diagnostic ability in large sample situations. Li, Huafeng et al. [5] proposed a denoising algorithm based on Contourlet transforms which can suppress Gaussian white noise. Although the problem of high image noise and low contrast in the infrared image fault detection technology of elevator traction machines has been solved, there are still shortcomings such as high cost, which cannot become a conventional method for identifying operating conditions. Wang Y et al. [6] studied the application of the least squares support vector machine regression algorithm in the fault prediction of the traction machine, and proposed a method for automatically searching the optimal parameters, which optimized the parameters and avoided the blindness of artificial selection. However, the support vector machine algorithm is only suitable for second-class classification and is difficult to implementation for large-scale training samples [7].

In the field of fault diagnosis, Mayeda et al. [8] developed a fault diagnosis method for elevator traction machines based on fuzzy neural networks and improved genetic algorithms. The vibration signal and temperature signal are used to train the model, and the improved genetic algorithm is used for feature selection and parameter optimization. Zhu et al. [9] proposed a deep learning based fault diagnosis method for elevator traction machines. Convolutional neural networks (CNN) and long short-term memory (LSTM) neural networks are used to extract and classify the features of vibration signals in order to achieve accurate fault diagnosis. Zhou et al. [10] proposed an intelligent diagnosis method for elevator traction machines based on a hybrid feature selection method. The research uses the vibration signal collected by the vibration sensor and the temperature signal collected by the temperature sensor, and combines mutual information and fuzzy clustering methods to select the most representative features for fault diagnosis. These studies were conducted under the premise of malfunctions and did not pay attention to the speed changes during normal equipment operation.

Liu Y. et al. [11] used a new demodulation method based on time–frequency analysis and principal component analysis (DPCA) to extract periodic modulated wave signals, which provided a new method for the rapid and effective condition monitoring of traction machinery. This method can be extended to detect other background disturbances and typical faults. Based on the above reasons, in response to the operating status of the traction machine during idle operation, data were obtained by arranging vibration sensors to monitor the operating status of the traction machine at 6 different speeds. This paper presents a novel approach that combines the demodulation method based on principal component analysis (DPCA) with the visual geometry group network (VGG) model in CNN, aiming to identify the operational state of the traction machine. The innovation of this method lies in its ability to not only achieve state recognition of traction machine vibration signals but also exhibit strong demodulation performance even under low signal-to-noise ratio conditions. By integrating DPCA with the VGG model, the proposed approach enhances feature extraction from vibration signals and improves the accuracy of identifying the running state of the traction machine. In addition, the results were compared with other algorithm models to verify the superiority of the proposed DPCA-VGG16 state recognition model. Finally, a conclusion was drawn.

## 2. DPCA-VGG16 State Recognition Model

**Figure 1:**
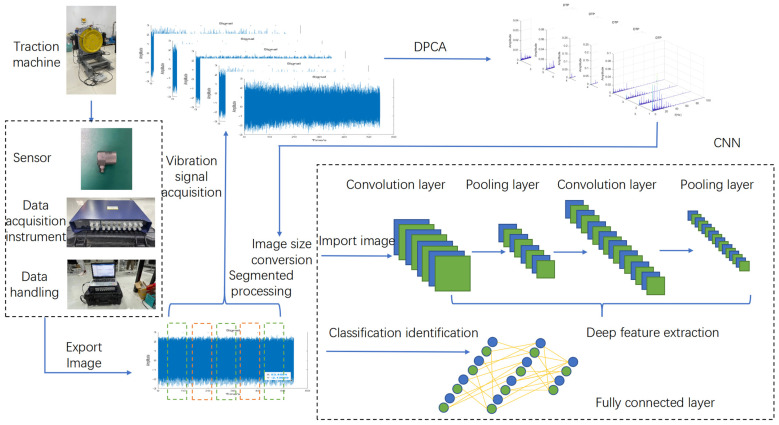
DPCA-VGG16 state recognition model.

This model demodulates vibration experimental test signals under different operating states to obtain demodulation graphs and then inputs the graphs under different states into the VGG16 structural model [12]. Through training and testing, the goal of automatic recognition of different operating states is achieved as shown in Figure 1.

### 2.1. DPCA Algorithm

The DPCA method is a signal feature extraction method based on time-frequency analysis and principal component analysis [13]. The basic principle of this algorithm is to first perform a short-term Fourier transform on the collected signal, determine the frequency and phase of the local sine wave of the time-varying signal, and obtain a time-frequency map. After that, calculate the time-frequency distribution matrix through time-frequency analysis. Then, principal component analysis is used to obtain the low-frequency characteristic modulation frequency of the signal, which is the principal component of the measured signal. Finally, the modulated signal components obtained are expanded in the frequency domain using a fast Fourier transform.

The time-frequency analysis uses short time fourier transform (STFT). Firstly, the signal is divided into several segments and windowed for each segment. Typically, the overlapping length of the signal is smaller than the length of the windowed signal. Then, for each segmented signal after windowing, a fast fourier transform (FFT) is performed to obtain signal features in the frequency [14]. Finally, the frequency domain signals obtained from each segment are concatenated to obtain the time-frequency spectrum of the entire signal. The horizontal axis represents time, the vertical axis represents frequency, and the color depth represents the strength of the signal at that time and frequency.

The STFT of a continuous time signal is shown in formula (1):

(1) $$X(\tau ,\omega )=\int_{-\infty }^{+\infty }x(t)w(t-\tau )e^{\left(-j\omega t\right)}dt$$

Among them, x(t) is the original signal, w(t−τ) is the window function, τ is the segmented time, and ω is the frequency.

The window function is used to divide the signal into multiple time periods for spectral analysis. Common window functions include rectangular windows, Hanning windows, Blackman windows, etc. [15]. Among them, the Hanning window can reduce the frequency domain leakage problem, and its amplitude attenuation is smoother than other windows. Therefore, it is selected as the window function of this algorithm. Hanning’s mathematical expression is shown in formula (2):

(2) $$\omega (n)=0.5-0.5\cos\left(\frac{2\pi n}{N-1}\right)$$

Among them, w(n) represents the window coefficient of the *n*th sampling point, and *N* is the window length.

The amplitude spectral density function of the modulated signal refers to that of a periodic non-random signal. The amplitude spectral density function of the modulated signal is calculated within the range that meets the sampling theorem and the minimum frequency limit, which can improve the efficiency of the algorithm. the calculation of the time-frequency distribution function of a single component modulated signal is shown in formula (3). This function describes the distribution of signal amplitude in the frequency domain.

(3) $$P(f,t)=\frac{2\times \left|P_{x}(f,t)\right|}{L_{\mathrm{FFT}}}$$

The results are combined into a time-frequency distribution matrix for principal component analysis [16], as shown in formula (4).

(4) $$P(t,f)=\left[\begin{array}{cccc} P\left(t_{1},f_{t}\right) & P\left(t_{2},f_{t}\right) & \cdots & P\left(t_{n},f_{t}\right)\\ P\left(t_{1},f_{t}+\Delta f\right) & P\left(t_{2},f_{t}+\Delta f\right) & \cdots & P\left(t_{n},f_{t}+\Delta f\right)\\ \vdots & \vdots & \vdots & \vdots \\ P\left(t_{1},f_{m}\right) & P\left(t_{2},f_{m}\right) & \cdots & P\left(t_{n},f_{m}\right) \end{array}\right]$$

The time-frequency distribution matrix reveals the variation patterns of signals in both time and frequency dimensions, providing a basis for subsequent principal component analysis and feature extraction [17].

Principal Component Analysis (PCA) is a classic data dimensionality reduction method commonly used for processing, feature extraction, and visualization of high-dimensional data [18]. After time-frequency analysis, principal component analysis can be performed on the time-frequency distribution matrix of a single component modulated signal to extract its most significant time-frequency information, thereby obtaining the low-frequency characteristic modulation frequency of the modulated signal. Through the covariance function, the time-frequency distribution matrix is converted into the covariance matrix as shown in formula (5) [19].

(5) $$P_{\cot\;}=\mathrm{cov}(P(t,f))$$

To decompose the covariance matrix into a diagonal matrix, we can perform an eigenvalue decomposition. The process involves obtaining the eigenvalues and eigenvectors of the covariance matrix.

We can extract key feature information from the signal by decomposing the covariance matrix of a single component modulated signal. The algorithm primarily focuses on obtaining the eigenvalues and corresponding eigenvectors. The selection of eigenvalues follows the principle of the difference spectrum, as depicted in formula (6). Energy features and information about the entropy features play a significant role in the selection process. The order of eigenvalues is determined by the maximum value of the difference spectrums.

(6) $$k\geq {\left.i\right|}_{\max\left(\varepsilon_{1}-\left(\lambda_{1}-\lambda_{1}+1\right)\right.}$$

The principal component reconstruction method is employed to derive the corresponding principal component of the modulation signal. This technique relies on principal component analysis, which involves mapping the original data into a lower-dimensional space while preserving as much information from the original data as possible.

The procedure begins by conducting principal component analysis on the eigenvalue-decomposed feature vectors to obtain the principal components and their respective weight coefficients. Next, a desired number of principal component fractions to be retained is selected. Based on these weight coefficients, the original feature vectors are mapped into a lower dimensional space. By utilizing the inverse transformation of the weight coefficients, the corresponding principal component modulation signal is obtained.

Finally, the modulated signal undergoes a transformation from the time domain to the frequency domain using a Fast Fourier Transform (FFT).

### 2.2. VGG16 Structure

CNN is an exemplary deep feedforward artificial neural network that draws inspiration from biological receptive mechanisms. It typically comprises a convolutional layer, a pooling layer, and a fully connected layer [20]. The convolutional layer aims to extract image features, while the pooling layer’s purpose is to simplify the extracted features [21]. Once the convolutional and pooling layers generate new feature vectors, they are commonly mapped through a nonlinear function to enhance the expressive capacity that linear operations may lack.

The principle of CNN is to construct multiple filters that can extract features from input data. These filters convolute and pool the input data layer by layer, gradually extracting structural features hidden in the data [22]. As the network structure deepens, the extracted features gradually become more abstract, ultimately obtaining input data feature representations that are invariant to translation, rotation, and scaling. CNN incorporates several techniques, such as: sparse connections, weight sharing, and spatial or temporal downsampling. Sparse connections establish non-fully connected spatial relationships between layers, reducing the number of trainable parameters. This helps prevent overfitting of the model and efficiently captures important spatial information. Weight sharing is employed to share the same set of weights across different spatial locations, enabling the network to learn generalizable patterns and improve computational efficiency. Subsampling involves downsampling the spatial or temporal dimensions of the data. By exploiting the local features and characteristics inherent in the data, subsampling reduces data dimensionality, optimizes the network structure, and ensures a certain degree of invariance to displacement. Therefore, CNN is highly suitable for processing and learning from massive amounts of data as it effectively captures complex and robust representations through its deep architecture and utilization of various techniques [23].

**Figure 2:**
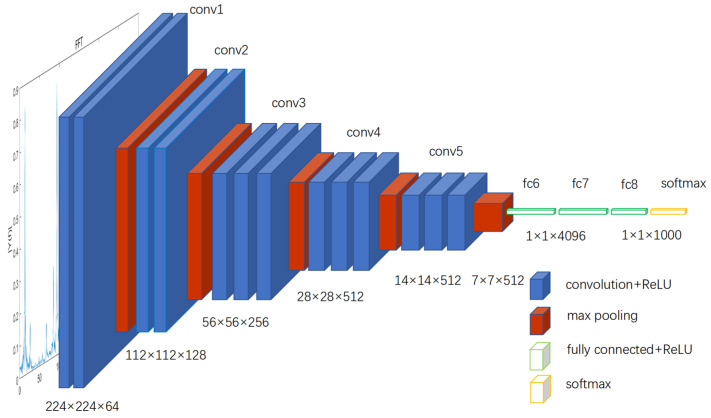
VGG16 Structure.

VGG16 is a common CNN structure, and its name comes from the abbreviation of the Visual Geometry Group. VGG16 is a deep Convolutional Neural Network (CNN) composed of 13 convolutional layers and three fully connected layers, totaling 16 layers in total. The network employs multiple small-sized (3 × 3) convolution kernels for the convolution operation. Following each convolution layer, a Rectified Linear Unit (ReLU) activation function is applied to enhance the expressive power of non-linear features. Additionally, VGG16 utilizes pooling layers to reduce the size of feature maps [24]. Before the final fully connected layer, a flattening operation is applied to convert the 3D feature map into a one-dimensional vector. Classification is then performed through three fully connected layers [25]. The structure of the VGG16 network consists of 13 convolutional layers, five pooling layers, and three fully connected layers, as illustrated in Figure 2 [26].

Compared with other CNN structures, such as LeNet and AlexNet, VGG16 network is deeper, but it also brings some problems, such as gradient disappearance, overfitting, etc. [27]. Furthermore, VGG16 maintains a uniform size for the convolution kernels, employs smaller strides, and utilizes padding layers. These design choices contribute to a simpler network architecture, striking a good balance between computational efficiency and the number of model parameters [28].

## 3. Design of the Experimental Platform

**Figure 3:**
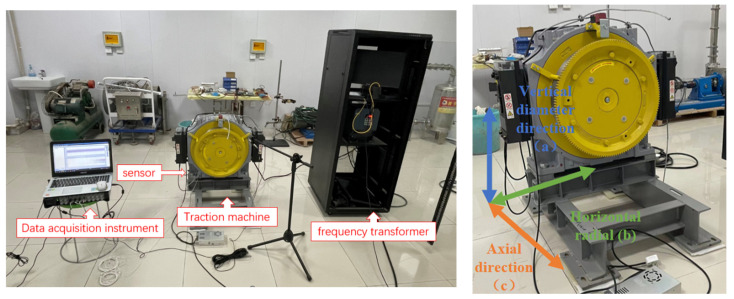
Experimental system.

To verify the effectiveness of the model proposed in this article, a traction machine state recognition experimental platform was designed to collect the vibration signals of the traction machine at six different rotational speeds. After data processing, the vibration signals were used as inputs to the DPCA-VGG16 model, and a traction machine state recognition model was constructed. The test data comes from the traction machine test bench, which was mainly composed of the traction machine, universal vector inverter, acquisition instrument, vibration acceleration sensor, etc., as shown in Figure 3.

The traction system utilized the magnetic Vito-200/1000 permanent magnet synchronous gearless traction machine, with its parameters listed in Table 1. The machine comprised essential components such as the stator, rotor, and base, which were energized by permanent magnets. To enhance control over the start and stop operations of the traction machine, separate turning gear switch components and brakes were installed.

To ensure the diversity of experimental data, three directions were selected to obtain vibration data. As shown in Figure 3, the vibration acceleration sensor was placed on the base of the traction machine and fixed along the vertical diameter, as well as the horizontal, radial, and axial directions using a magnetic base. The sensor parameters are shown in Table 2. The running direction of the traction machine was clockwise and upward, without any load. To ensure the effectiveness and safety of the experiment, data sampling was selected during the stable operation stage of the traction machine, with a steady-state sampling time of no more than 60 s and an input frequency of 5–30 Hz. Extreme ultra-high and ultra-low frequencies were removed to achieve efficient classification. According to formula (7), at the maximum input frequency of 30 Hz, the main vibration frequency was only 112.5 Hz. Therefore, the sampling frequency of the dynamic signal was set to 600 Hz. This included most harmonic frequencies. If a higher resolution was chosen, it would be affected by high-frequency signals, which would affect the resolution of the spectrum and frequency domain.

(7) n=60f/p

## 4. Experiment and Analysis

### 4.1. Dataset Feature Extraction

**Figure 4:**
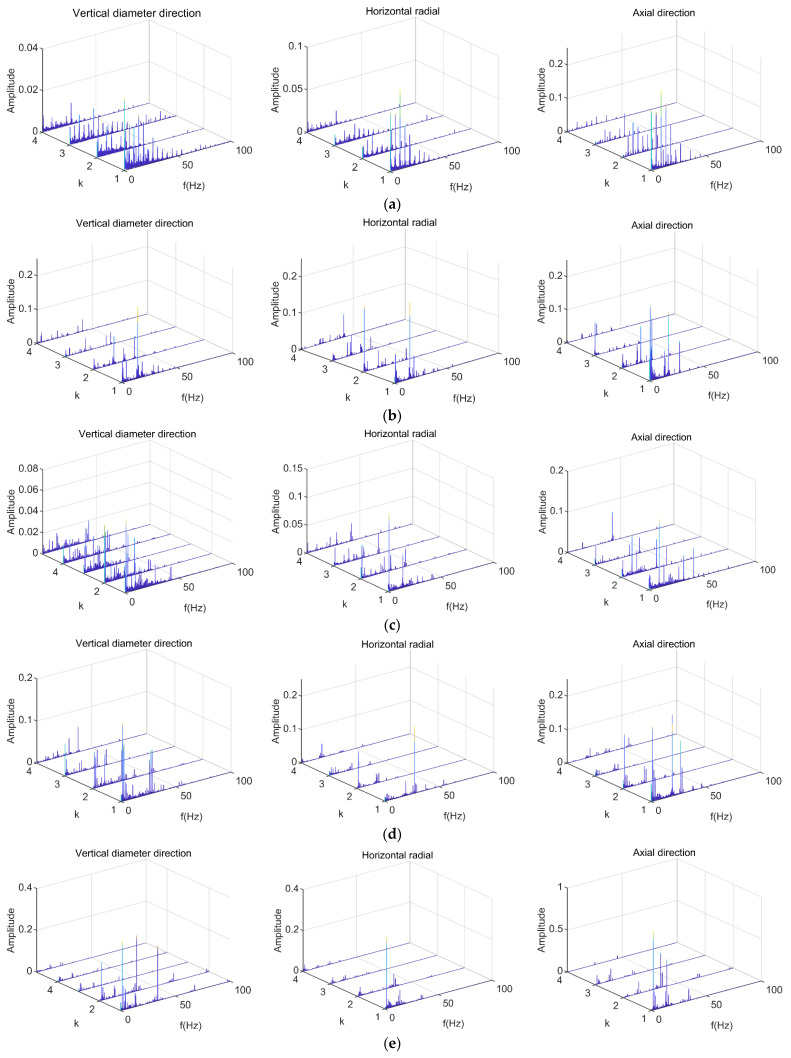

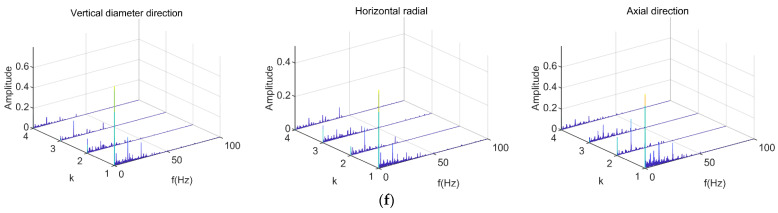
Signal demodulation diagram. (**a**) Input frequency is 5 Hz; (**b**) Input frequency is 10 Hz; (**c**) Input frequency is 15 Hz; (**d**) Input frequency is 20 Hz; (**e**) Input frequency is 25 Hz; (**f**) Input frequency is 30 Hz.

Based on the above collection conditions, vibration signals of elevator traction machine equipment can be obtained under six different operating states. These operating states all operate at a steady speed without load, but their input frequencies are different. The input frequencies are 5 Hz, 10 Hz, 15 Hz, 20 Hz, 25 Hz, and 30 Hz, respectively. The vibration signals were input into the DPCA demodulation algorithm to obtain the demodulation diagram. The three principal components of the vibration signals were selected for feature extraction. The demodulation results obtained are shown in Figure 4, where the directions represented from left to right are: vertical diameter direction, horizontal radial direction, and axial direction.

Following the demodulation process, the harmonic component of the signal tended to be relatively small in magnitude. However, significant harmonic components were still present. Although there may have been some variations in the spectral distribution and amplitude across the three directions, the main frequencies corresponding to the same operating state remained largely consistent. However, direct analysis through the demodulation spectrum requires sufficient experience and there are certain errors, which is not conducive to forming standardized diagnostic logic and rapid recognition under big data input. In addition, although the fundamental vibration frequency of the traction machine can be determined, due to the limitations of the testing environment, the features of image samples not recognized by CNNs were not obvious, including some vibration clutter and low-frequency noise that cannot be avoided in experiments. When high input frequencies were applied, the vibration frequency detected in the frequency spectrum may not have been lower than the vibration frequency observed in the demodulation map generated from low input frequencies. These differences cannot be easily discerned by visual inspection alone. Further image processing is required to extract features and utilize machine learning techniques for identification and classification tasks.

The paper proposes to verify the DPCA-VGG16 state recognition model using different operating status signals of the traction machine. The verification process involves several steps outlined below:

(1) Divide the vibration signal data into equal-length segments (200 segments) over time.
(2) Perform DPCA demodulation on each segment, converting the time-domain diagram into a demodulation frequency domain diagram.
(3) Group all the demodulated images into 6 categories based on different input frequencies, with each group containing 200 image samples.
(4) Randomly select 160 image samples as the training set and 40 image samples as the test set, ensuring that the same algorithm model performs consistently on different sets by shuffling the data before loading it.
(5) After applying the VGG16 model, continue fitting the data, drawing accuracy and loss curves.

The image input size was 224 × 224. This is because the convolutional layer and pooling layer structures in the VGG16 model were designed for image sizes of 224 × 224. The batch size was set to 128. This can make the calculation amount of each batch moderate, fully utilize the parallel computing power of the GPU, and avoid the problem of insufficient graphics memory. At the same time, larger batch sizes can also lead to a smaller update range, so it was possible to reduce the vibration of the model in the training process, thus improving the rate of convergence and stability of the model. The epoch was set to 20. It can enable the model to obtain sufficient learning and optimization during the training process, without causing overfitting or excessive training time issues. The validation split was set to 0.2. A portion of the data in the training set can be used as a validation set for evaluation in order to better evaluate the model’s generalization ability. Using this method can avoid the impact of model differences caused by random initialization and other reasons, and can also avoid overfitting problems when evaluating the use of test sets. The initial learning rate was set to 0.1, making the model converge quickly, and then the learning rate was gradually reduced to improve the model performance. Weight decay was set to 0.0001. This controls the strength of the regularization term to help avoid overfitting. The dropout rate set to 0.3. This randomly inactivated some neurons in the network, which helped prevent overfitting.

In order to avoid bias and instability caused by unreasonable dataset partitioning and effectively utilize limited datasets, cross-validation of the model was conducted. It should be noted that when using cross-validation, it is necessary to ensure that the partitioning method of the dataset and the hyperparameter settings of the model are the same in order to obtain reliable evaluation results.

### 4.2. Result Analysis

**Figure 5:**
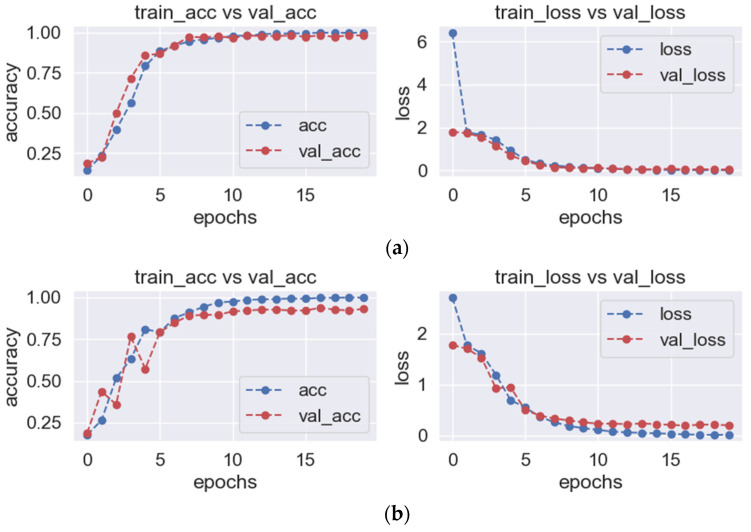

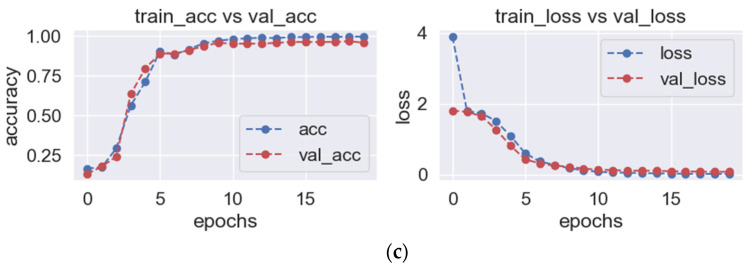
Accuracy and Loss Curve. (**a**) Vertical diameter direction; (**b**) Horizontal radial; (**c**) Axial direction.

As shown in Figure 5, it can be observed that as the training frequency increases, the training accuracy improves and the loss decreases in all three directions. The specific data changes are provided in Table 3. The results indicate that the model achieves the highest recognition performance for vibration data with a vertical upward direction, achieving a validation rate of 100% after 10 training sessions. However, due to the relatively small differentiation in the horizontal, radial, and axial directions, more training sessions are required to achieve convergence. In the early stages of the machine learning process, the accuracy rate for the horizontal radial direction may decline. This can be attributed to the limitations of the algorithm, as manually set hyperparameters such as the number of layers, nodes, and learning rate may not perfectly match all sample data. However, with the increase in training times, it can converge quickly, and the accuracy of the training set eventually approaches 100%. It can be seen that the model has a high generalization ability in identifying the status of the traction machine, and the model can automatically predict and efficiently judge the operation status of the traction machine in a short period of time.

**Figure 6:**
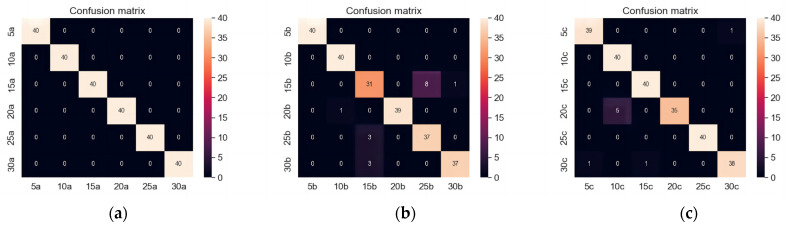
Confusion matrix of operation status identification results. (**a**) Vertical diameter direction; (**b**) Horizontal radial; (**c**) Axial direction.

Based on Figure 6, the confusion matrix illustrates the operation status recognition results under different input frequencies. The recognition success rate for the vertical diameter direction is 100%. For the horizontal radial direction, the recognition success rate is 93.75%, while for the axial direction, it is 97.08%. Overall, the comprehensive recognition success rate is calculated to be 96.94%. These results indicate that the DPCA-VGG16 model achieves high accuracy in recognizing the operation status of the traction machine, with the vertical diameter direction yielding the highest recognition success rate. The results indicate that compared to axial and horizontal radial directions, the experimental data in the vertical diameter direction are more comprehensive and accurate in the operation status of the traction machine.

**Figure 7:**
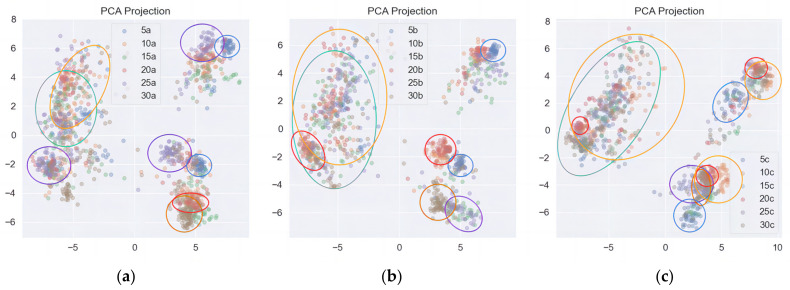
PCA dimensionality reduction diagram of operating state recognition results. (**a**) Vertical diameter direction; (**b**) Horizontal radial; (**c**) Axial direction.

To gain a more concrete understanding of sample features and to visualize them, PCA (Principal Component Analysis) dimensionality reduction was performed on the image samples. This process reduces the 4-dimensional features to 2-dimensional ones. The results of dimensionality reduction are displayed in Figure 7.

PCA not only compresses data into lower dimensions but also ensures that the features of the reduced data are independent of each other. From the visualization, it can be observed that the samples with an input frequency of 5 Hz are clearly distinguished in all three testing directions. The image features in the vertical diameter direction exhibit higher separability compared to the horizontal and axial images, where the separability is relatively poor. As the input frequency increases, the distinguishability of the samples gradually decreases.

### 4.3. Contrast Test

**Figure 8:**
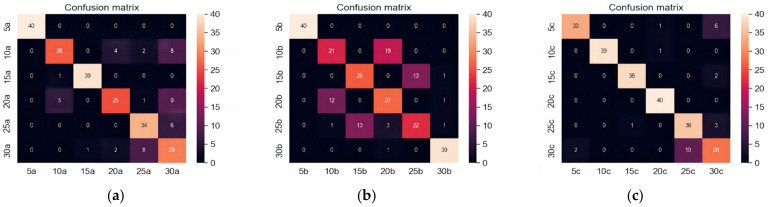
Confusion matrix identified by the time domain diagram. (**a**) Vertical diameter direction; (**b**) Horizontal radial; (**c**) Axial direction.

In order to verify the correlation between the DPCA algorithm and model recognition, the original time-domain data map was exported and divided into equal time periods. Time domain images were drawn using time as the horizontal axis and signal amplitude as the vertical axis to display the changes in the signal in the time dimension. Time-domain images were trained with the same parameters as the training for the DPCA demodulation images to control variables. 80% of all image samples were placed in the training set and 20% in the test set. The average success rate of recognition was 80.33%, as shown in Figure 8:

Accuracy and recall are commonly used metrics for evaluating models. As shown in Table 4, accuracy represents the ratio of correctly predicted samples by the classifier to the total number of samples. The DPCA-VGG16 model exhibits higher accuracy compared to the time domain VGG16 model, indicating its superior classification ability and more accurate data categorization.

Table 5 describes the recall rate, which refers to the proportion of correctly predicted positive cases by the classifier to the actual number of positive cases. The DPCA-VGG16 model achieves a higher recall rate than the time domain VGG16 model, demonstrating its enhanced capability in recognizing positive cases and accurately identifying true positive instances. The average success rate of the time domain image input VGG16 recognition model is 80.33%. The test results in all three directions are not as good as the test results after the DPCA demodulation transformation of the time-domain image, indicating that the image features of the time-domain image are not prominent enough compared to the demodulated image. DPCA demodulation transformation is a necessary image transformation process.

**Figure 9:**
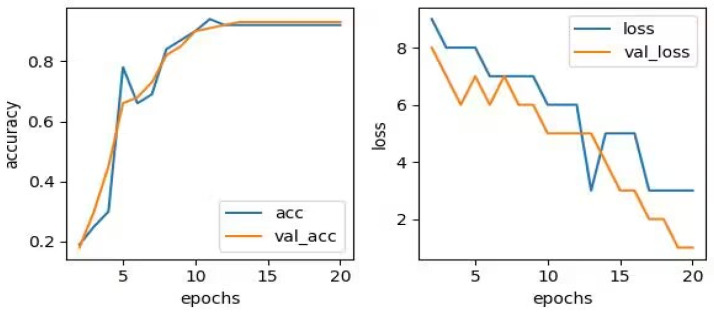
Resnet Network Accuracy and Loss Curve.

In addition to VGG16, commonly used CNN models also include AlexNet, ResNet, etc. [18]. The ResNet network has a deeper structure, consisting of 152 convolutional layers. Due to the continuous multiplication operation required for gradient backpropagation, error signals may be multiplied multiple times, resulting in very small gradients, making it difficult for the network to learn deeper features. Moreover, the training time of the ResNet model is too long, which is not conducive to the recognition of operational status under a large amount of data. The recognition success rate of inputting images processed by the DPCA algorithm into the AlexNet network is only 92.50%. The accuracy and loss diagram of the Resnet network is shown in Figure 9.

**Figure 10:**
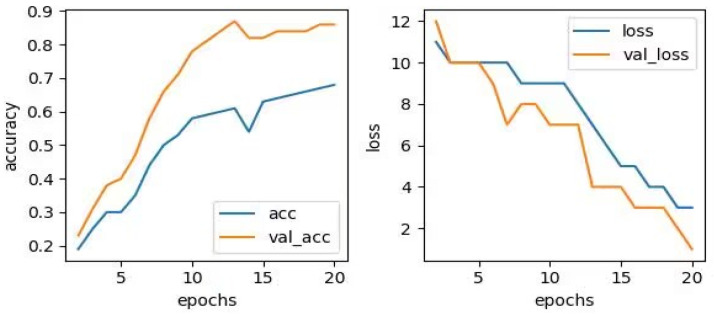
Alexnet Network Accuracy and Loss Curve.

The AlexNet network has a shallow depth of only 8 layers and differs from VGG16 in terms of convolutional kernel size and stride. In AlexNet, the convolutional kernel and pooling kernel sizes are not constant but vary according to the needs of specific layers. Specifically, the first five layers of AlexNet are convolutional layers, with the first layer using 11 × 11 convolutional kernels, four-pixel stride, and 96 filters; layer 2 using 5 × 5 convolutional kernels, two-pixel stride, and 256 filters; layers 3, 4, and 5 using 3×3 convolutional kernels, a one-pixel stride, and 384, 384, and 256 filters, respectively. After the convolutional layer, there are three pooling layers, each using 2 × 2 pooling kernels and a two-pixel stride. The recognition success rate of inputting images processed by the DPCA algorithm into the AlexNet network is only 68.33%. Compared to the VGG16 model, its recognition accuracy is significantly insufficient. The accuracy and loss diagram of the Alexnet network is shown in Figure 10.

## 5. Conclusions

The vibration signals of the traction machine contained a large amount of effective information that reflects its operating status. The DPCA-VGG16 recognition model was proposed to address the problem of identifying the operating status of the traction machine and was based on the DPCA and CNN. The vibration features were extracted from the traction machine and acquired by the acquisition platform. The proposed DPCA-VGG16 recognition model achieved operation status recognition. Through research, the following conclusions have been drawn:

(1) The DPCA-VGG16 traction machine state recognition model was proposed with a recognition accuracy of 96.94%. It is better to directly import the time-domain graph into the convolutional network model. The comprehensive recognition accuracy, training duration, and feasibility are superior to recognition models such as AlexNet, GoogleNet, and ResNet.
(2) According to the given information, it can be inferred that the signal characteristics of traction machine vibrations are most prominent in the vertical diameter direction, resulting in stronger recognition performance. The axial direction exhibits relatively clear signal characteristics as well, while the horizontal and radial directions have weaker signal characteristics.

By focusing on measuring the vibration signals in the vertical diameter direction, the DPCA-VGG16 model is better equipped to accurately identify the operating status of the traction machine. This suggests that the vertical diameter direction provides more distinct and informative features for effective state recognition.

## Figures and Tables

**Table 1:** Traction Machine Parameters.

Parameter	Value
Weight/kg	381
Traction ratio	2:1
Number of poles/P	32
Rated power/kW	13.4
Rated load/kg	1000
Rated frequency/Hz	51
Rated speed r/min	191
Rated voltage/V	340
Rated current/A	30.2
Efficiency/%	85.1

**Table 2:** Sensor Parameters.

Test Direction	Vertical Diameter Direction (a)	Horizontal Radial (b)	Axial Direction (c)
Model	352C33	352C33	352C33
Sensitivity	10.23	10.38	9.94

**Table 3:** Accuracy and loss of the traction machine operation status identification.

Number of Training Sessions in Data Direction (Times)	Vertical Diameter Direction		Horizontal Radial		Axial Direction	
	Accuracy (%)	Loss	Accuracy (%)	Loss	Accuracy (%)	Loss
3	39.69	1.6599	52.73	1.5050	29.17	1.7333
5	79.37	0.9291	75.91	0.7251	71.35	1.0825
10	96.77	0.1274	95.44	0.1595	97.14	0.1187
20	100	0.0051	100	0.0166	99.74	0.0142

**Table 4:** Accuracy rate of traction machine status recognition under the same operating condition.

Data Direction Input Frequency (Hz)	Vertical Diameter Direction		Horizontal Radial		Axial Direction	
	DPCA-VGG16	Time Domain vgg16	DPCA-VGG16	Time Domain vgg16	DPCA-VGG16	Time Domain vgg16
5	100	100	100	100	97.5	94.29
10	100	81.25	97.56	61.76	88.89	100
15	100	97.5	83.78	66.67	97.56	97.44
20	100	80.65	100	55.10	100	95.24
25	100	75.56	82.22	62.86	100	78.26
30	100	55.77	97.37	92.86	97.44	71.79

**Table 5:** Recall rate of traction machine status recognition under the same operating condition.

Data Direction Input Frequency (Hz)	Vertical Diameter Direction		Horizontal Radial		Axial Direction	
	DPCA-VGG16	Time Domain vgg16	DPCA-VGG16	Time Domain vgg16	DPCA-VGG16	Time Domain vgg16
5	100	100	100	100	100	82.5
10	100	65	100	52.5	100	97.5
15	100	97.5	77.5	65	100	95
20	100	62.5	97.5	67.5	87.5	100
25	100	85	92.5	55	100	90
30	100	72.5	92.5	97.5	95	70

## Data Availability

Data can be accepted on request.
